# An Uncommon Complication in an Unlikely Patient: Vaping-Induced Spontaneous Pneumomediastinum in a Young Female Patient

**DOI:** 10.7759/cureus.88559

**Published:** 2025-07-22

**Authors:** Otioli Wambalaba, FNU Manisha, Sarah Abubakr

**Affiliations:** 1 Department of Internal Medicine, Griffin Hospital, Derby, USA; 2 Department of Internal Medicine, Peoples University of Medical and Health Sciences for Women, Nawabshah, PAK; 3 Department of Internal Medicine, Buffalo General Medical Center, Buffalo, USA

**Keywords:** differential diagnosis of acute chest pain, electronic cigarette-associated lung injury, macklin effect, spontaneous pneumomediastinum (spm), vaping-associated pneumomediastinum

## Abstract

We report the case of a 21-year-old underweight female patient with generalized anxiety disorder (GAD) who presented to the Emergency Department with acute chest pain and dyspnea. She had no significant past medical history aside from GAD, regular vaping, and marijuana use. Her clinical evaluation revealed stable vital signs and unremarkable labs aside from mildly elevated troponins. A CT pulmonary angiogram identified a pneumomediastinum. She was admitted and observed overnight. As her condition remained stable, she was managed conservatively with oral acetaminophen and bowel regimen, counselled on vaping cessation, and discharged with outpatient follow-up. Post-discharge, she remained asymptomatic, and repeat surveillance imaging demonstrated the resolution of pneumomediastinum.

This case underscores the need for high clinical suspicion of vaping-associated pneumomediastinum (VAPM) in patients who vape and develop new chest symptoms. This is especially important in atypical populations and individuals with anxiety-related disorders. We add to the limited literature of female presentations of VAPM and reinforce the importance of increased awareness among clinicians.

## Introduction

Vaping-associated pneumomediastinum is a rare but increasingly recognized condition linked to the use of e-cigarettes. E-cigarettes were patented in 2003 and introduced to the US market in 2007 [1,2]. The use of e-cigarettes among adults has increased from 4.5% in 2019 to 6.5% in 2023, with young adults being the most prevalent users [3]. This translates to about 1.98 million young adults. Increase in usage brought about an increase in diagnosis of electronic cigarette-associated lung injury (EVALI) and other well-documented respiratory tract-related injuries [4]. One of more rare complications is vaping-induced spontaneous pneumomediastinum (VAPM). The clinical course of uncomplicated VAPM is usually benign, and the management is supportive. However, there is potential for life-threatening complications such as tension mediastinum, cardiac tamponade, and pneumopericardium, which require monitoring [5]. Symptoms typically include acute chest pain and difficulty breathing, while imaging studies confirm the presence of air in the mediastinum [5,6].

Spontaneous pneumomediastinum (SPM) is traditionally a process that is more prevalent in young, thin, and tall men [7]. This pattern may also be seen in VAPM. Out of the six previously reported cases of e-cigarette-associated pneumomediastinum, only one was a woman [6].

## Case presentation

Our patient is a 21-year-old woman with generalized anxiety disorder (GAD) who presented with a one-day history of acute-onset shortness of breath and sharp chest pain that worsened with deep breathing. The pain was non-radiating, and she denied fever, palpitations, cough, nausea, vomiting, loss of consciousness, or similar symptoms in the past. She works as a waitress, vapes daily, drinks alcohol on weekends, and smokes marijuana. The initial differential diagnosis included pulmonary embolism, pneumonia, pneumothorax, esophageal tear, vaping-associated lung injury, pericarditis, myocarditis, costochondritis, and anxiety.

Physical examination

Her initial blood pressure (BP) was 110/73, heart rate 98, and respiratory rate 21, and she was afebrile, saturating at 100% on room air. She was 5'3'', 98 pounds with a BMI of 17.4 kg/m2. On examination, she was not in distress. Her chest revealed bilaterally equal air entry, without crepitus or jugular venous distension.

Labs

Her lab workup revealed no leukocytosis. Influenza and COVID tests were negative. Her troponins were elevated at 44 but trended downward to 41 (Table 1). EKG showed no significant abnormalities. She was given a HEART Score of 1 based on her elevated troponins, indicating low risk of a major cardiac event. Her elevated troponins were attributed to demand ischemia requiring no further inpatient cardiac workup.

**Table 1:** Relevant labs taken on admission HGB: hemoglobin; WBC: white blood cell; SARS-CoV-2: severe acute respiratory syndrome coronavirus 2; RT-PCR: reverse transcriptase polymerase chain reaction

Test	Result		Reference range
HGB	14.6		12.0-16.0 g/dL
WBC	9.0 x 10^9^		(4.8-10.8) x 10^9^/L
High-sensitivity D-dimer	<150		0-243 ng/mL
High-sensitivity troponin I	44.64	41.39	Female: <34.0 ng/L
Influenza A (rapid)	Not detected		Not detected
Influenza B (rapid)	Not detected		Not detected
SARS-CoV-2 RNA (RT-PCR)	Not detected		Not detected
Urine marijuana screen	Positive		Negative

Imaging

**Figure 1:**
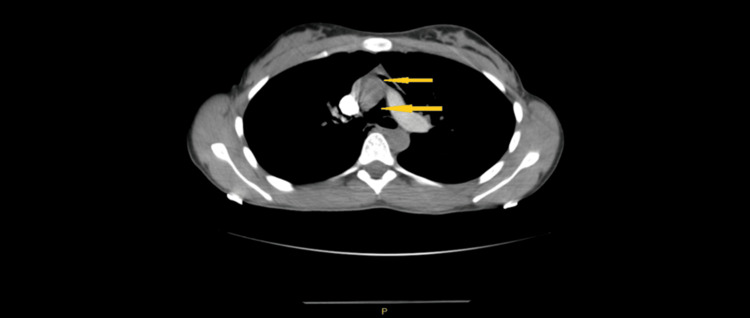
CT pulmonary angiogram showing the pneumomediastinum Yellow arrows indicate air in the mediastinum

CT pulmonary angiogram showed pneumomediastinum, predominantly in the superior aspect, tracking into the right lower neck. No pulmonary embolism, pericardial effusion, hilar or mediastinal lymphadenopathy, or right heart strain was noted (Figure 1).

Hospital course

The patient was observed overnight on telemetry and managed with oral acetaminophen and stool softeners, leading to the resolution of her symptoms. The Cardiothoracic Surgery service assessed her the following morning and recommended discharge with symptomatic care and outpatient follow-up. Additionally, she was provided counseling on vaping cessation. No management of her anxiety was offered as her baseline symptoms had been well controlled and there did not appear to be a psychiatric component contributing to her presentation. She was given a referral to Pulmonology, who ordered a repeat non-contrast CT chest, which demonstrated complete resolution of the pneumomediastinum.

## Discussion

VAPM is a rare but increasingly recognized complication of electronic cigarette use, especially in young adults. Traditionally, SPM has been most commonly reported in tall, thin men [5]; however, the rising popularity of vaping may be changing the typical patient profile. Of the limited number of vaping-associated pneumomediastinum cases reported in the literature, only a small proportion have involved women, making our case a notable contribution [6]. The reasons for VAPM being less common in women are not fully understood but may be due to a combination of factors that are not fully elucidated in the literature. However, men have demonstrated a predominance when it comes to vaping and EVALI [8].

The pathophysiology of vaping-associated pneumomediastinum is understood to involve the Macklin effect, whereby alveolar rupture allows air to dissect along the bronchovascular sheaths into the mediastinum [9]. This process typically results from elevated intrathoracic pressures caused by forceful inhalation, breath-holding, or coughing-mechanisms commonly associated with vaping and marijuana use [10]. Unlike pneumomediastinum due to trauma or infection, the air leak in these cases occurs without direct structural injury to the airways or esophagus and is primarily driven by pressure changes [9].

This case contributes to the growing body of evidence linking e-cigarette use to pneumomediastinum and highlights the importance of clinician awareness, early diagnosis, and counseling on the risks of vaping, even in the absence of overt trauma or underlying lung disease. Specifically, inquiring about the history of vaping is crucial. Although the clinical course of SPM and VAPM is usually regarded as benign, they do have the potential to develop into rare life-threatening conditions [5,9].

## Conclusions

This case of a 21-year-old female patient with GAD, history of vaping, and no other classic risk factors highlights potential shifting demographics of VAPM. Her hospital course entailed one day of observation on telemetry, supportive care consisting of oral analgesics, and review by Cardiothoracic Surgery. She was discharged home after maintaining stable vitals and resolution of symptoms. She followed up with Pulmonology and repeat CT chest that demonstrated the resolution of the pneumomediastinum.

Clinicians should maintain a high index of suspicion for VAPM in patients with unexplained chest pain and a history of vaping, even in the absence of trauma or underlying lung disease. Early diagnosis, supportive care, and counseling on vaping cessation are essential in managing this condition and preventing further complications. Interventions targeting accessibility and appealability to the youth as well as addressing nicotine addiction may have the best long-term impact from a population health perspective.
